# The Impact of Perceived Teacher Emotional Support on Student Learning: A Meta-Analysis

**DOI:** 10.3390/bs16081322

**Published:** 2026-08-03

**Authors:** Xi Fu, Xiaoyong Zhou

**Affiliations:** School of Foreign Languages, East China Normal University, Shanghai 200241, China; 52290400009@stu.ecnu.edu.cn

**Keywords:** meta-analysis, teacher emotional support, academic achievement, student engagement, academic self-efficacy

## Abstract

Teacher emotional support (TES) has been widely recognized as an important factor influencing students’ learning and development. However, empirical studies provide inconsistent evidence regarding the strength of the relationship between those two. The present meta-analysis, therefore, synthesized studies published between 2000 and 2025 to estimate the overall association between TES and three key dimensions: academic self-efficacy, student engagement, and academic achievement. Results revealed a significant positive association between TES and student learning. Specifically, TES demonstrated relatively stronger effects on academic self-efficacy (r = 0.463) and engagement (r = 0.456) than on academic achievement (r = 0.259). Moderator analyses further revealed variation by several contextual factors, including educational level and discipline, with strongest associations observed among higher education students and in language-related disciplines. The findings highlight the importance of emotionally supportive teacher–student relationships for the learning process. The meta-analytical results provide important implications for educational practice and policy.

## 1. Introduction

The “affective turn” in education has highlighted the critical role of emotion and relational dynamics in learning (Clough, 2008; Dernikos et al., 2020; Hargreaves, 1997, 1998, 2000). Teacher emotional support (TES), defined as students’ perceptions of teachers’ consistent provision of care, empathy, encouragement, and interpersonal warmth, fosters psychological safety and relational trust in the classroom (Herath & Tejada-Sanchez, 2025; Tao et al., 2022).

TES is grounded in Self-Determination Theory (SDT; Deci & Ryan, 2000), which emphasizes the satisfaction of basic psychological needs (relatedness, competence, and autonomy), and the Self-System Process Model, which proposes that perceived teacher support nurtures students’ academic self-efficacy. This, in turn, promotes engagement and, more distally, academic achievement (Olivier et al., 2019; Pierson & Connell, 1992). In the present meta-analysis, TES is operationalized strictly as students’ perceived emotional care and warmth, as measured by validated instruments targeting the affective-relational dimension of teacher–student interactions. This focus distinguishes TES from broader teacher support constructs that also include instrumental or informational elements.

Empirically, TES has been linked to three key dimensions of student learning: academic self-efficacy (Bandura, 1977, 1986, 1993; L. He et al., 2024), student engagement (Fredricks et al., 2004; Fredricks et al., 2016; Skinner & Belmont, 1993), and academic achievement typically operationalized as test scores, grades, and GPA (White, 1982). Although positive associations are frequently reported, findings remain inconsistent in strength and significance across studies (e.g., Davis, 2001; Gorman et al., 2002; Han et al., 2022), suggesting context-dependent effects.

Several prior meta-analyses have examined related constructs. Roorda et al. (2011) synthesized affective teacher–student relationships but not TES specifically and found medium-to-large links with engagement and smaller links with achievement. Tao et al. (2022) reported a small-to-medium overall correlation between broader perceived teacher support and achievement (r = 0.16), with engagement as a partial mediator, but did not isolate the emotional dimension or include self-efficacy as a core outcome. Vargas-Madriz et al. (2024) focused on different domains of teacher support and dimensions of school engagement while excluding self-efficacy and achievement. J. Liu et al. (2024) examined social support and undergraduate achievement without distinguishing TES or addressing engagement.

None of these syntheses systematically integrated all three learning dimensions (academic self-efficacy, engagement, and achievement) under the specific construct of perceived TES, nor did they comprehensively examine the full range of contextual moderators (educational level, geographic region, discipline, learning mode, and student type). The present meta-analysis addresses these gaps by synthesizing studies published between 2000 and 2025 from both English- and Chinese-language databases. It estimates the overall association between TES and student learning, compares effects across the three dimensions, and tests key moderators.

The present study addresses these gaps through a meta-analytic synthesis of empirical studies on TES and student learning. Specifically, it pursues the following research questions:

RQ1: What is the overall association between perceived TES and student learning?

RQ2: Does the strength of this association differ across the three learning dimensions—academic self-efficacy, student engagement, and academic achievement?

RQ3: Do educational level, geographic region, discipline, learning mode, and student type moderate the association between TES and student learning?

## 2. Literature Review

### 2.1. TES and Academic Self-Efficacy

Academic self-efficacy refers to students’ judgments of their ability to succeed in academic tasks (Bandura, 1977, 1986, 1993). Such beliefs promote motivation, persistence, and performance (L. He et al., 2024). Perceived TES consistently strengthens academic self-efficacy by enhancing students’ sense of competence and control. This association appears across contexts, including American middle schools (Sakiz et al., 2012), Chinese elementary and college EFL classrooms (R. Liu et al., 2018; Wang et al., 2023), and online learning (Duan et al., 2024). Effect sizes vary, however, indicating potential contextual influences (Kitsantas et al., 2021; Kong et al., 2025; Skaalvik et al., 2015).

### 2.2. TES and Student Engagement

Student engagement is a multidimensional construct that includes behavioral, emotional, cognitive, agentic, and social components linking classroom contexts to learning outcomes (Fredricks et al., 2004; Reschly & Christenson, 2022; Skinner & Belmont, 1993; Wong & Liem, 2022). TES plays a central role in fostering engagement by creating a psychologically safe classroom climate (Furrer & Skinner, 2003; Patrick et al., 2007; Reeve, 2013; Wentzel, 2009). Positive associations have been found for behavioral (T. Huang & Liu, 2025; Ruzek et al., 2016), emotional (Cesnaviciene et al., 2022; Sakiz et al., 2012), cognitive (Pineda-Báez et al., 2019; A. M. Ryan & Patrick, 2001), and agentic engagement (Chiu, 2022; Pineda-Báez et al., 2019) in secondary, mathematics, and EFL settings. Some studies, however, report non-significant links, particularly for behavioral engagement among elementary or low-SES students (Bingham & Okagaki, 2012; Cesnaviciene et al., 2022), suggesting moderation by developmental and contextual factors.

### 2.3. TES and Academic Achievement

Academic achievement, typically measured by test scores, course grades, and GPA, is generally positively associated with TES across educational levels (Baker, 2006; Brock et al., 2008; Klem & Connell, 2004; Kosir & Tement, 2014; Perry et al., 2007). Yet findings are mixed: some studies show non-significant direct effects (Han et al., 2022; C. Huang et al., 2023) or primarily indirect influences through motivational mediators (Jensen et al., 2019; Ruzek et al., 2016). This pattern aligns with theory, indicating that TES is more strongly associated with proximal outcomes (self-efficacy and engagement) than with the more distal outcome of academic achievement.

### 2.4. Potential Moderators

The strength of TES-learning associations likely varies by context. Potential moderators include educational level (Hamre & Pianta, 2001; Roorda et al., 2011; Ulmanen et al., 2023), geographic region (reflecting cultural differences in teacher authority and relational norms; Cortina et al., 2017; X. Sun et al., 2019; N. Zhou et al., 2012), academic discipline (Cheng et al., 2023; Curby et al., 2009; Erdem & Kaya, 2024), learning mode (online vs. offline; Jiang et al., 2021; Vagos & Carvalhais, 2022), and student type (e.g., immigrant, ethnic minority, or left-behind students; Abdulhamed & Beattie, 2024; Den Brok et al., 2010; Ramazan et al., 2023). Existing evidence for these moderators remains inconsistent, warranting systematic meta-analytic investigation.

### 2.5. Research Gap

Prior meta-analyses have addressed related questions but leave important gaps. Roorda et al. (2011) examined broader affective teacher–student relationships and their links to engagement and achievement, without isolating TES. Tao et al. (2022) investigated perceived teacher support (including emotional aspects) and its relations to engagement and achievement (with engagement as a mediator), but did not focus exclusively on the emotional dimension or include self-efficacy. Vargas-Madriz et al. (2024) analyzed different domains of teacher support and dimensions of school engagement while excluding self-efficacy and achievement. J. Liu et al. (2024) focused on social support dimensions and undergraduate achievement without distinguishing TES or examining engagement.

None of these syntheses systematically integrated academic self-efficacy, engagement, and achievement under the specific construct of perceived TES, nor did they comprehensively test the full set of contextual moderators (educational level, geographic region, discipline, learning mode, and student type) included here. Addressing these gaps, the present meta-analysis synthesizes 64 studies yielding 83 effect sizes from English- and Chinese-language databases (2000–2025) to estimate the overall association between TES and student learning, compare effects across the three dimensions, and examine key moderators.

## 3. Methodology

### 3.1. Literature Search Process

The literature search process was conducted systematically across both English and Chinese databases, including CNKI, Web of Science, and WILEY, yielding a total of 4224 records. Web of Science was used as the primary multidisciplinary database, while WILEY and CNKI were searched as complementary sources to enhance retrieval coverage and to include relevant Chinese-language studies. A combination of the key terms included (“emotional scaffolding” OR “affective scaffolding” OR “teacher emotional support” OR “teacher affective support” OR “teacher support”) AND (“learning achievement” OR “academic achievement” OR “learning outcomes” OR performance OR “self-efficacy” OR “engagement” OR “student engagement”) and their corresponding Chinese keywords “教师情感支持”, “情感支持”, “课堂情感环境”, “学习成就”, “学业成就”, “学习成果”, “学业表现”, “学习参与” and “自我效能”. The search process was carried out on 31 December 2025 and included articles published between January 2000 and 31 December 2025. After cross-checking titles and abstracts of articles, duplicates, non-journal articles and non-English or non-Chinese results (*n* = 725) were removed, leaving 3499 articles for further scrutiny.

### 3.2. Inclusion and Exclusion Criteria

In order to guarantee the relevance and quality of studies, the following criteria were applied: (a) studies examined the association between TES and academic self-efficacy, engagement and academic achievement; (b) studies employed quantitative methodology; (c) TES was treated as an independent variable. Studies labeled as ‘teacher support’ were also eligible if the measurement instruments exclusively assessed teacher emotional support, regardless of the terminology used by the original authors. To determine eligibility, the content of each measurement instrument, including the reported scale descriptions and, where available, the original items, was carefully examined. Only studies in which the instrument exclusively captured emotional support (e.g., warmth, care, respect, emotional encouragement, responsiveness, or sensitivity) rather than instructional, academic, or instrumental support were retained; and (d) studies reported sufficient statistical information, including sample size and correlation coefficients, to compute effect sizes.

**Figure 1:**
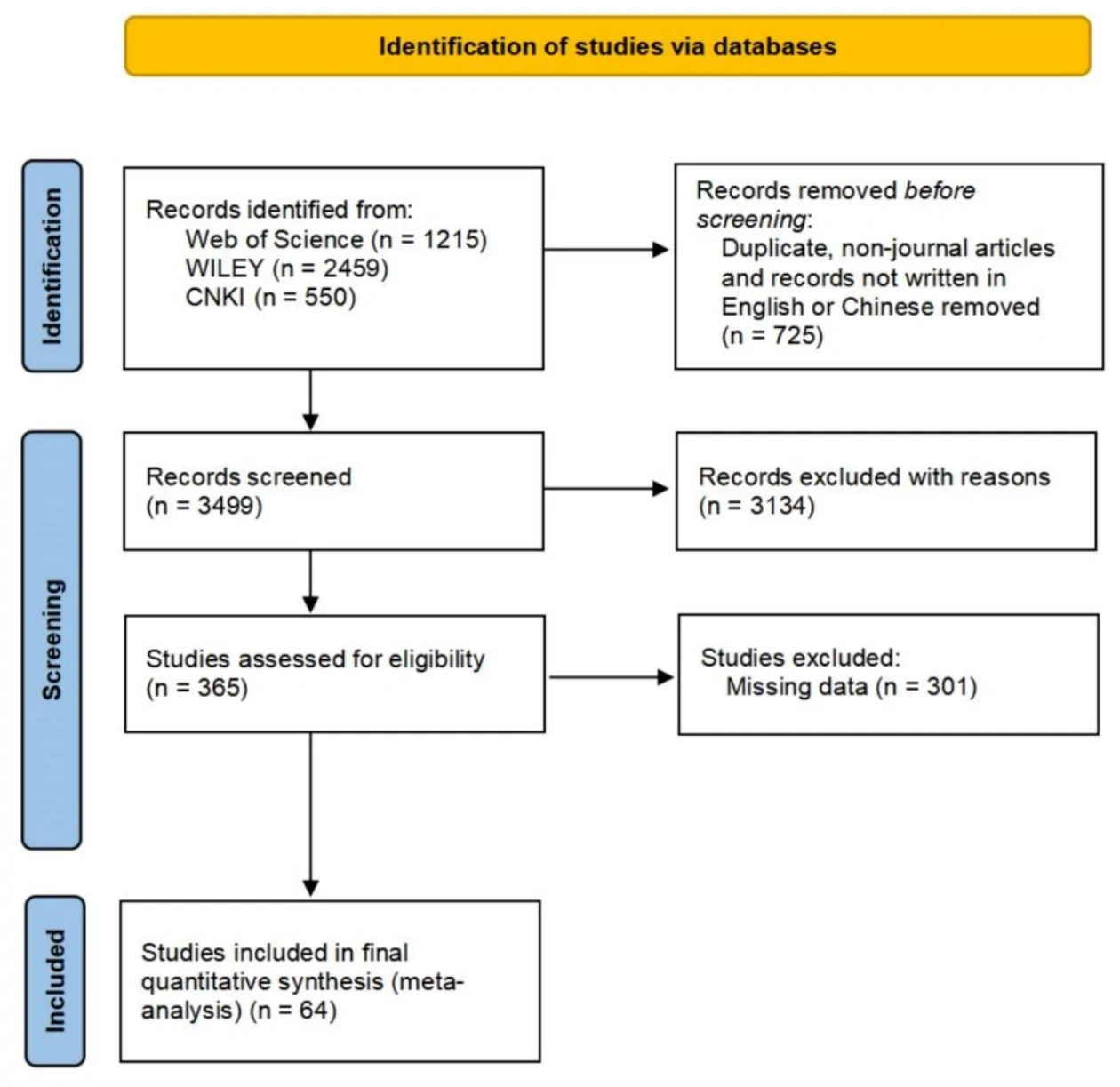
PRISMA flowchart of literature search process.

Based on these criteria, the full texts of 365 eligible articles were further examined. A total of 301 studies were excluded due to two reasons: either they lacked sufficient statistical information for effect size computation (e.g., missing or non-convertible correlation coefficients), or they failed to isolate an operational measure of perceived teacher emotional support from broader constructs (e.g., general teacher support). The final sample comprised 64 articles, from which 123 effect sizes were extracted for coding and subsequent meta-analytic analyses (see Figure 1).

### 3.3. Coding of Studies

To account for within-study dependence and avoid the underestimation of standard errors, effect sizes extracted from the same independent sample were aggregated into a single study-level size prior to meta-analysis (Borenstein et al., 2009b). The data extraction and coding process followed a rigorous two-stage collaborative protocol to ensure accuracy. The first author conducted the complete data extraction and coding for all included studies, producing the dataset presented in Appendix A. The included articles were coded for the following variables: (a) study number (No.), (b) study name, (c) sample size, (d) educational level, (e) geographic region, (f) discipline, (g) type of student learning outcomes, (h) student type (special vs. general population), (i) learning mode (online vs. offline), and (j) Pearson correlation coefficients (r). The substantive characteristics were categorized based on a standardized classification criterion (see Table 1). To evaluate coding reliability, the corresponding author double-coded a random 20% sample of the studies in Appendix A against the primary sources. The inter-rater agreement for the statistical data extractions was 98%, with minor clerical typos resolved via consensus.

### 3.4. Statistical Analyses

For studies that report a correlation between two continuous variables, the correlation coefficient (r) can serve as the effect size index (Borenstein et al., 2009a). All included studies reported the correlation coefficients between the perceived teacher emotional support and indicators of student learning. Accordingly, r was adopted as the statistical indicator of effect size. During the data processing, the correlation was converted to Fisher’s z scale. All analyses were performed using the transformed values and then converted back to the original metric (Borenstein et al., 2009a). To transform correlation r to Fisher’s z:

(1) $$z=0.5\times ln\left(\frac{1+r}{1-r}\right)$$

To convert Fisher’s z back to a r index:

(2) $$r=\frac{e^{2z}-1}{e^{2z}+1}$$

Within-study aggregation was conducted prior to the overall meta-analysis, with the within-study correlation among dependent effect sizes conservatively assumed to be ρ = 0.50 (Borenstein et al., 2009b). This procedure reduced the number of independent effect sizes included in the analyses from 123 to 83. To analyze all data, Comprehensive Meta-Analysis Software V3.0 (CMA) was used to merge effect sizes. Since observed effect size differences among studies include both real variations and random sampling error, heterogeneity testing was conducted using the Q statistic and I^2^ statistic to identify and quantify the variance from spurious variance (Borenstein et al., 2009a). Significant Q values (*p* < 0.05) indicate true heterogeneity beyond sampling error, while I^2^ on the order of 25%, 50%, and 75% represent low, moderate, and high heterogeneity (Higgins et al., 2003). The heterogeneity test results (Table 2) indicated high heterogeneity (Q = 2995.815, *p* < 0.01; I^2^ = 97.263%) across studies, leading to the adoption of a random-effects model to interpret the effect-size variability among different studies.

### 3.5. Publication Bias

**Figure 2:**
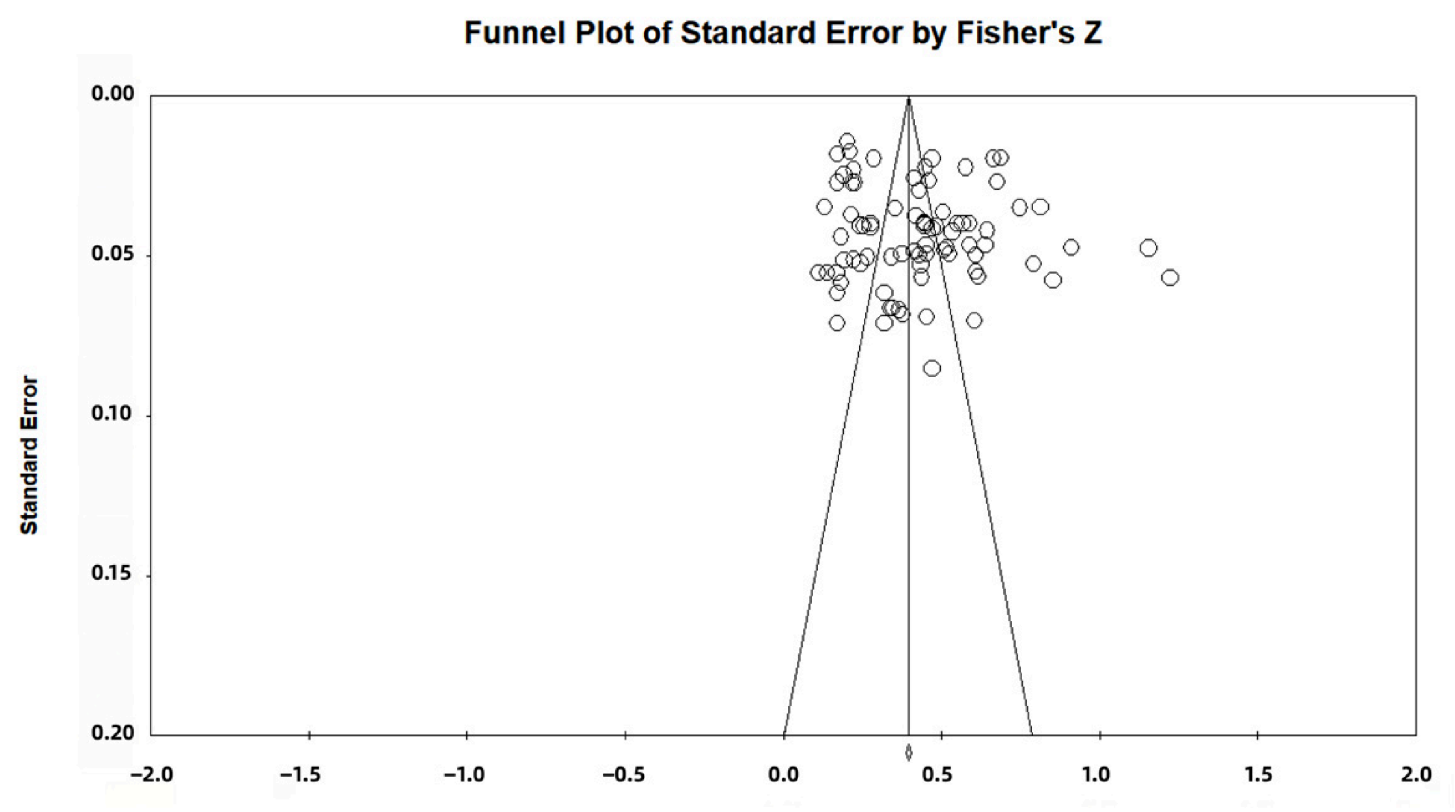
Funnel plot of standard error by Fisher’s Z. Circles represent the aggregated effect sizes from the included studies. The diamond represents the pooled effect size. The solid lines indicate the expected 95% confidence limits.

**Figure 3:**
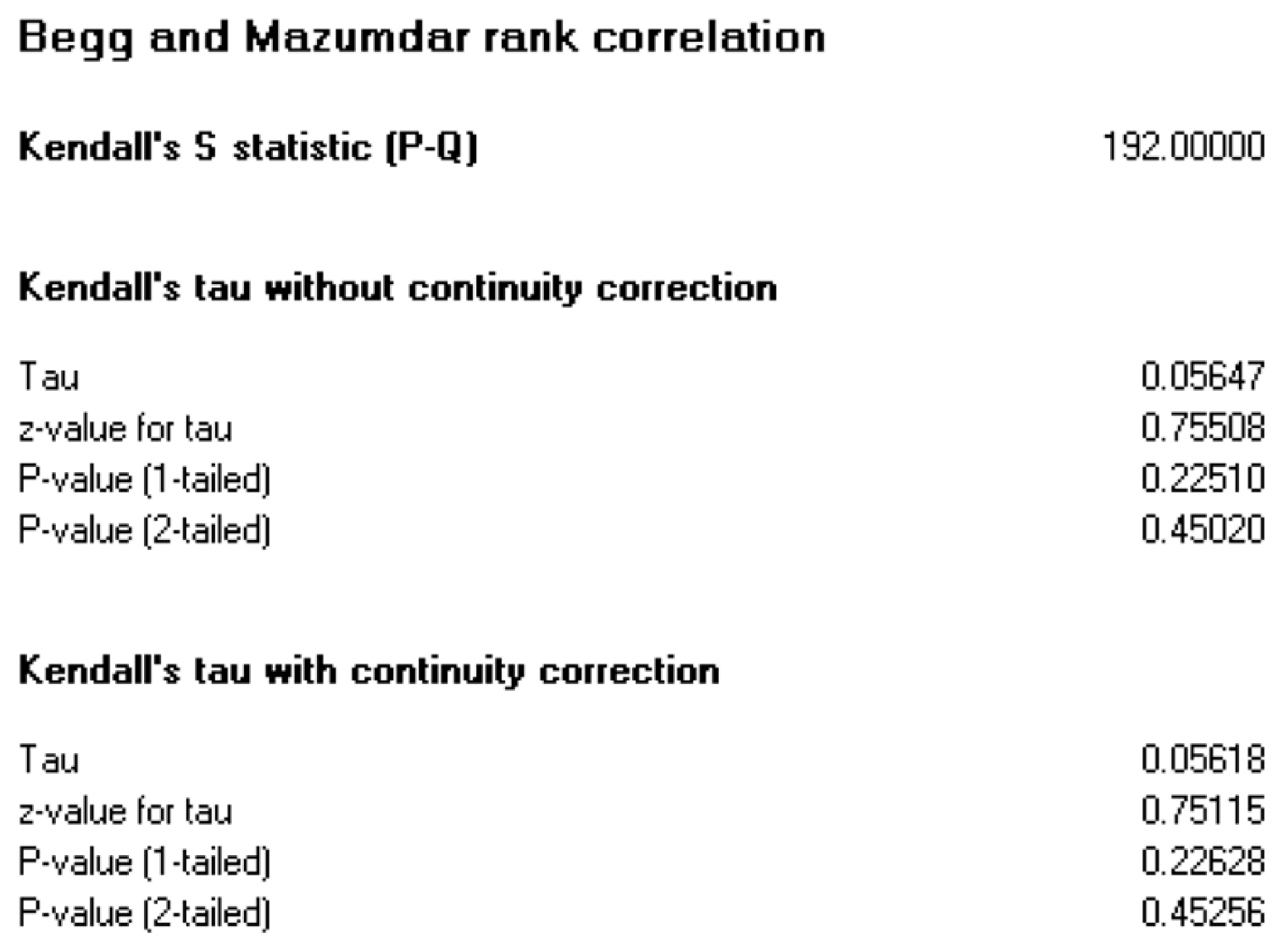
Begg’s and Mazumdar rank correlation results.

**Figure 4:**
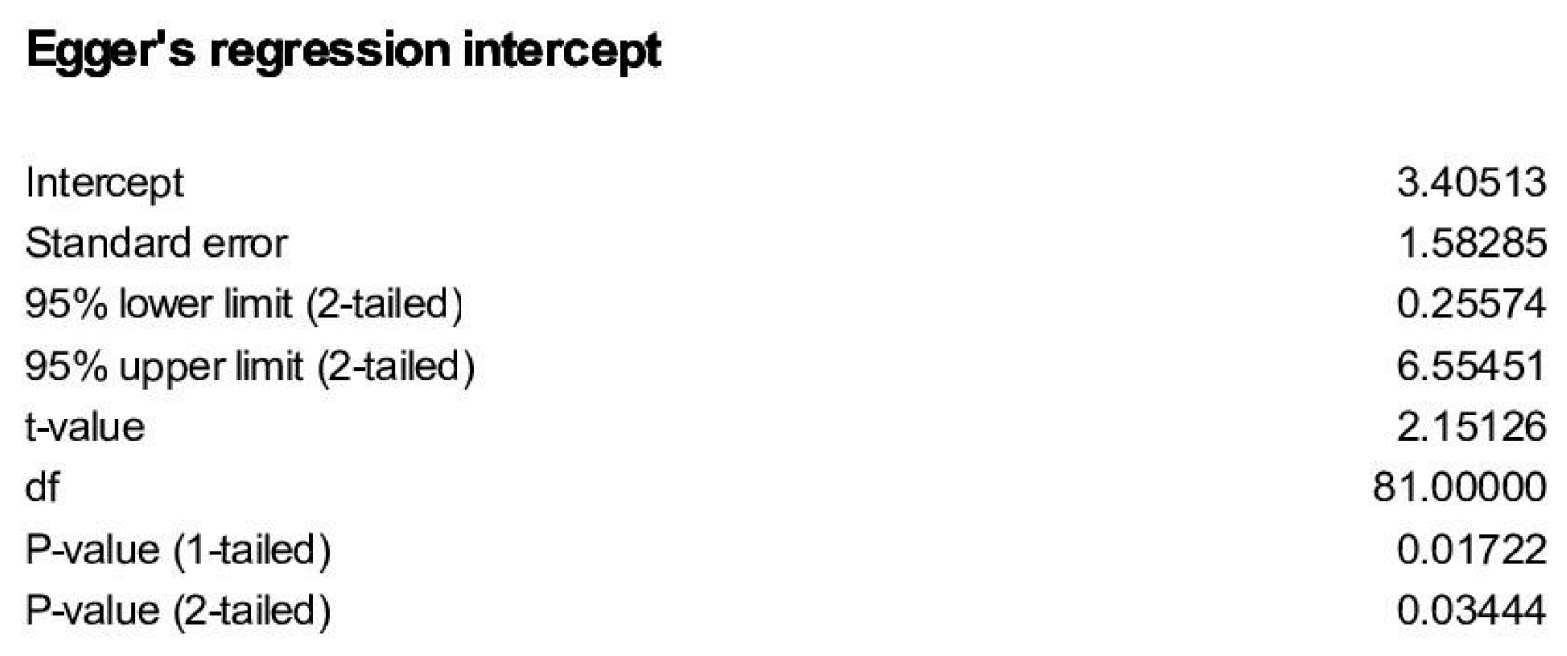
Egger’s regression intercept results.

**Figure 5:**
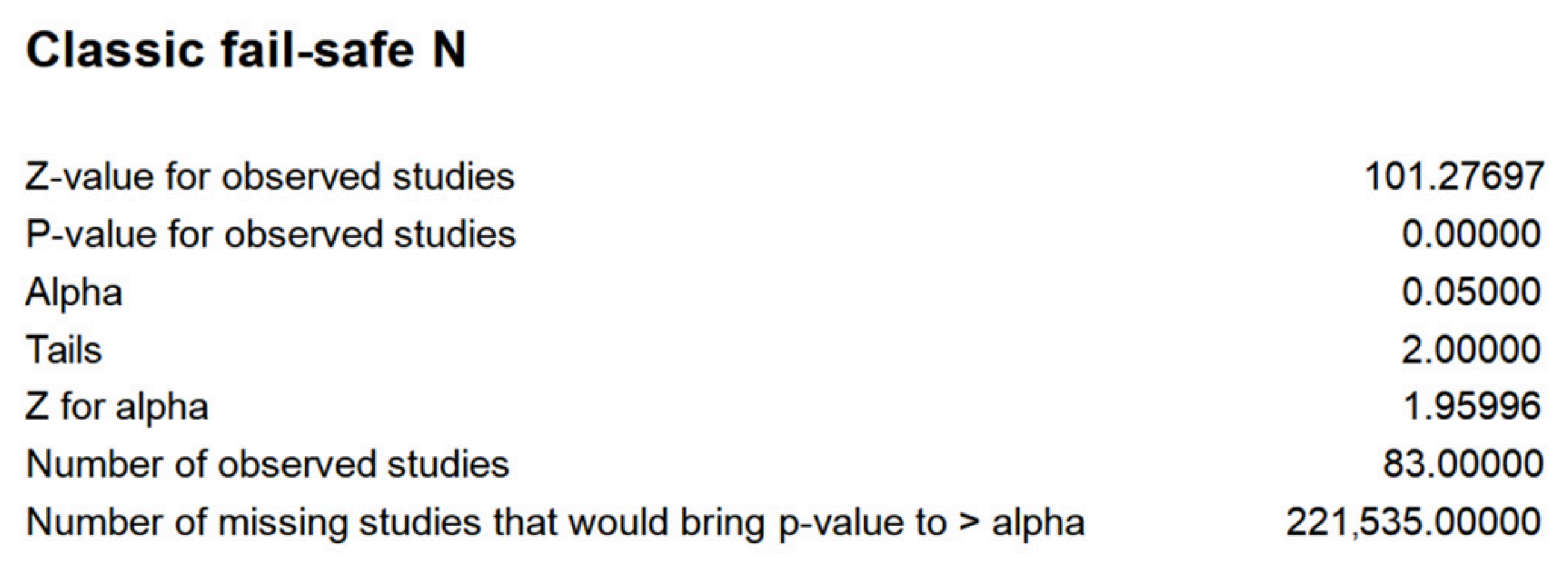
Classic fail-safe N analysis results.

Publication bias refers to the phenomenon that studies with statistically significant results, effect magnitudes and directions, and large study sizes are more likely to be accepted and published, potentially biasing meta-analytic estimates (Borenstein et al., 2009c; Vevea et al., 2019). Therefore, a funnel plot, Begg’s rank correlation test, Egger’ s Regression test, Duval and Tweedie’s trim-and-fill method, and the classic fail-safe N analysis were employed to detect the publication bias in this study. As shown in Figure 2, 83 effects were basically symmetrically distributed on both sides of the top of the funnel, indicating a low risk of publication bias. However, the results of Begg’s test (Figure 3) and Egger’s test (Figure 4) yielded conflicting results (*p* = 0.453 and *p* = 0.0344, respectively). Because substantial between-study heterogeneity was observed in the present meta-analysis (I^2^ = 97.263%), the significant Egger’s test may reflect heterogeneity rather than publication bias alone (Borenstein et al., 2009c). To further evaluate the robustness of the findings, the trim-and-fill method was applied (Duval & Tweedie, 2000). Under the random-effects model, the trim-and-fill analysis imputed 15 missing studies to the right of the mean effect size and zero study to the left, indicating that the observed pooled effect was a conservative estimate and resilient to publication bias. Finally, the classic fail-safe N was 221,535 (Figure 5), far exceeding the threshold of 5k + 10 (where k = 83), indicating that a large number of null-result studies would be required to attenuate the observed effect to non-significance (Rosenthal, 1979). Overall, the available evidence suggests that publication bias is unlikely to have materially affected the pooled effect estimate. Nevertheless, the significant result from Egger’s test should be interpreted with caution, as the substantial between-study heterogeneity may also have contributed to the observed asymmetry.

### 3.6. Sensitive Analysis

To further examine the robustness of the pooled effect, a one-study-removed sensitivity analysis was conducted using CMA. The results indicated that the pooled effect sizes remained highly consistent when each study was removed individually, ranging from r = 0.398 to r = 0.409, compared with the overall effect size of r = 0.406. These findings suggest that no single study exerted a substantial influence on the overall effect estimate.

## 4. Results

### 4.1. Descriptive Findings

The meta-analysis included 64 independent studies yielding 83 effect sizes. Because some studies reported more than one learning outcome, the number of effect sizes exceeded the number of studies. Specifically, the dataset consisted of 18 effect sizes for academic self-efficacy, 42 for student engagement, and 23 for academic achievement (Table 3). Study participants were drawn from primary, secondary, and higher education levels across 13 countries, representing East Asian, Western, and Middle Eastern contexts.

### 4.2. Effect Size Analysis

The meta-analysis yielded a significant overall effect size (r = 0.406, 95% CI [0.368, 0.443], see Table 2). R quantifies both the direction (positive/negative association) and the magnitude (strength of association) between variables. According to Cohen’s benchmarks (Cohen, 2013), the overall effect size indicates a moderate to large yet positive association between perceived teacher emotional support and student learning. Subgroup analyses showed that TES was positively associated with academic self-efficacy (r = 0.463), engagement (r = 0.456), and academic achievement (r = 0.259) (see Table 3). The between-group difference was significant (Q-between = 29.253), indicating that the strength of associations varied significantly across student learning outcomes, with the strongest effect observed for academic self-efficacy. All reported effects are statistically significant (*p* < 0.05) unless noted otherwise.

### 4.3. Effects of Moderators

The heterogeneity across studies suggests that moderators may account for between-study differences. Therefore, a moderator analysis was conducted to determine whether educational level, geographic region, discipline, learning mode, and student type moderated the impact of TES on students learning (see Table 4). For the discipline moderator, the category “N/A” refers to the studies in which the instructional discipline was not explicitly reported or was not applicable because the learning context was not tied to a specific subject area.

#### 4.3.1. Significant Moderators

Two moderators were statistically significant: educational level and discipline. Educational level moderated the association between TES and students learning (Q-between = 33.814), with the largest effect in higher education (r = 0.516). Discipline further moderated the association (Q-between = 108.596), with stronger effects in language-related studies (r = 0.480) and education (r = 0.474) than in science (r = 0.461). Substantial variation in the number of studies across subgroups should be noted. Several statistically significant but sparsely populated subgroups were based on very limited evidence, including disciplines such as chemistry (k = 1), literature (k = 1), education (k = 2), science (k = 2). Given the extremely small number of effect sizes in several of these categories, these subgroup estimates should be interpreted as preliminary descriptive effect sizes, rather than robust inferential meta-analytic results. Therefore, these findings represent exploratory patterns that require further empirical validation.

#### 4.3.2. Non-Significant Moderators

In contrast, geographic region, learning mode and student type were not significant moderators. Geographic region did not significantly moderate the relationship between TES and student learning (Q-between = 2.902, *p* = 0.407), indicating that the positive association transcends geographic boundaries. Similarly, no significant moderating effects were observed for learning mode (Q-between = 0.785, *p* = 0.376) or student type (i.e., migrant students, ethnic minority students, left-behind students; Q-between = 1.661, *p* = 0.198). These results show that the positive impact of TES on student learning remains robust across both online and offline delivery modes, as well as for both special and general student samples.

## 5. Discussion

### 5.1. Average Effect Size

The current meta-analysis examined the relationship between perceived teacher emotional support and student learning, revealing a statistically significant and positive correlation between the two, with a moderate-to-large overall effect size (r = 0.406, 95% CI [0.368, 0.443]), consistent with prior empirical research (Furrer & Skinner, 2003; Jia & Cheng, 2024; Shih, 2008). Across learning indicators, the strongest association was observed for academic self-efficacy (r = 0.463), followed by student engagement (r = 0.456), and academic achievement (r = 0.259). This pattern aligns with theoretical accounts positioning self-efficacy as a proximal determination of learning process, which may subsequently translate into engagement and academic performance (Artino, 2012; Luo et al., 2023). The present findings are consistent with previous meta-analyses demonstrating positive associations between broader forms of teachers support and student learning outcomes (Tao et al., 2022; Vargas-Madriz et al., 2024).

### 5.2. Moderator Discussion

The present research further identified several potential factors that may shape the relationship between perceived TES and student learning, including educational level and discipline. Educational level, a significant moderator, showed stronger TES-student learning associations at higher levels of education. This finding is consistent with prior meta-analytic and empirical evidence suggesting that teacher support exerts a stronger influence on older students (Kitsantas et al., 2021; Roorda et al., 2011; Tao et al., 2022). As students progress to higher educational levels, academic demands intensify and learning becomes more self-regulated (Edisherashvili et al., 2022; Khiat, 2022). Emotional support from teachers, therefore, may help sustain students’ motivation, reduce academic stress, and facilitate the effective use of self-regulated learning strategies. In such contexts, TES may function as a key resource that supports self-regulated learning processes, thereby strengthening its association with student learning.

The second statistically significant moderator identified in the present meta-analysis was academic discipline, with the largest effect size observed in language-related domains. One plausible explanation is that language classrooms typically involve frequent interaction, communication, and opportunities for self-expression, where caring, trusting, and supportive teacher–student relationship help create a safe and supportive environment that encourages participation and reduces anxiety (Reyes et al., 2012; Xie & Derakhshan, 2021). Moreover, TES may facilitate language learning by fulfilling students’ basic psychological needs for autonomy, competence, and relatedness as conceptualized in Self-Determination Theory (Dincer et al., 2019). When these needs are satisfied, students may feel more confident in expressing their ideas and using the target language, with less fear or embarrassment about making mistakes (M. Zhou et al., 2025). This finding highlights the particularly important role of emotional support in interaction-intensive learning environments.

On the other hand, the non-significant moderation findings suggest that the association between TES and student learning may be relatively stable across geographic regions, different instructional settings, and student compositions. First, no evidence about the moderating role of geographic region has been observed, which implies that the positive role of teacher emotional support is evident across Western, East Asian, and Middle Eastern contexts. This finding is theoretically consistent with Self-Determination Theory, which posits that the need for relatedness is a universal basic psychological need whose satisfaction is essential for healthy development and well-being regardless of culture (Deci & Ryan, 2000). It also aligns with previous meta-analytical evidence demonstrating positive associations between teacher support and student outcomes (Tao et al., 2022), as well as empirical studies conducted in culturally diverse contexts, including Japan, Russia, China, Peru, Belgium, and USA (B. Chen et al., 2015; Deci & Ryan, 2000).

Second, the moderation analysis indicated that the relationship between TES and student learning did not differ significantly between online and offline teaching contexts. This finding suggests that the functional role of TES may not be inherently contingent on physical classroom presence. In other words, TES may remain effective even when instructions occur in a virtual environment. One possible explanation is that the mechanisms through which TES promotes learning, such as enhancing students’ sense of academic competence and learning confidence, and mitigating the weakening effect of learning burnout on academic self-efficacy, can operate across instructional modalities (G. Yang et al., 2022). Existing research consistently reports positive association between TES and various aspects of student learning in both online and offline settings (Engels et al., 2020; Fan, 2024; Feng et al., 2023; X. He & Wen, 2025). However, this moderation effect should be interpreted with caution, due to the relatively limited number of exclusively online samples included in the present meta-analysis.

Third, the meta-analytical findings suggest that the positive effects of TES on student learning may be relatively consistent across diverse student types. The absence of a moderating effect of student population may reflect the broadly shared nature of students’ basic psychological needs. According to Self-Determination Theory, support for basic psychological needs promote students’ wellness across age, ethnicity, and culture (R. M. Ryan & Deci, 2020). In education settings, emotionally supportive teachers may foster students’ sense of relatedness and belonging, thereby encouraging active participation in learning and strengthening their confidence in academic contexts (Furrer & Skinner, 2003). Because these socio-emotional processes are fundamental to learning rather than specific to particular student groups, the benefits of TES may remain relatively stable across diverse populations. This interpretation is further supported by empirical evidence demonstrating comparable associations between TES and student engagement and academic achievement among minority and majority students (Engels et al., 2020; Karakus et al., 2023). However, evidence remains limited regarding whether the association between TES and academic self-efficacy is equally robust across to different student populations, highlighting an important direction for future research.

### 5.3. Implication

The present meta-analysis provides quantitative evidence that emphasizes the importance of teacher emotional support in student learning. The findings indicate that TES is more strongly associated with students’ motivational and psychological processes (i.e., academic self-efficacy and engagement) than with academic achievement, indicating that its influence on performance may be more indirect and contingent on additional factors. The identified moderating effects further underscore that the strength of the association between TES and student learning varies across educational levels and disciplines, highlighting the importance of considering developmental and instructional contexts and adopting a context-sensitive perspective when examining and applying teacher–student interactions. The findings also provide several implications for educational practice. They point to the value of strengthening teachers’ socio-emotional competencies in pre-service education and in-service professional development. In classroom practice, fostering emotionally supportive teaching climate, through respecting students’ perspectives, and providing emotional feedback that encourages participation and confidence in learning, may facilitate students’ engagement, academic confidence, and ultimately their learning outcomes. Finally, at the policy level, the present findings suggest that TES should be considered as a component of instructional quality within specific educational contexts. The relatively stronger associations observed in higher education and language-related disciplines indicate that TES may play a particularly significant role in these contexts. Accordingly, policies aimed at promoting effective teaching practices may benefit from incorporating socio-emotional dimensions of instruction alongside cognitive and curricular considerations.

## 6. Conclusions

The present meta-analysis synthesized 83 effect sizes from 64 articles to examine the relationship between perceived teacher emotional support and student learning. Results indicated that TES is positively associated with academic achievement, engagement, and academic self-efficacy, with a moderate-to-large overall effect size. Educational level and discipline emerged as significant moderators of these associations, whereas geographic region, student type, and learning mode did not, suggesting that the benefits of TES are broadly robust across diverse educational contexts while varying in magnitude across instructional settings. These findings provide robust evidence that teacher emotional support constitutes an important socio-emotional component of effective teaching and consistently contributes to multiple dimensions of student learning. By synthesizing evidence across different educational contexts and learning outcomes, this meta-analysis extends the current understanding of the educational value of TES and highlights its relevance for both educational research and classroom practice.

Despite the contributions of the present study, several limitations should be taken into consideration. Firstly, the number of samples available for some subgroups was relatively limited, which may reduce the stability and generalizability of estimates. This is particularly relevant for primary school samples, certain disciplines, online or blended learning contexts, and specific student populations, such as ethnic minority and left-behind students. In addition, the discipline moderator should be interpreted with caution because a substantial proportion of the included studies could not be classified into a specific disciplinary category. Future studies should report disciplinary context more explicitly to facilitate more informative moderator analysis. Future meta-analyses could expand the evidence basing these areas to provide more stable estimates and a more comprehensive understanding. Secondly, most included studies employed cross-sectional designs, constraining the ability to examine changes in student learning over time. Longitudinal research is encouraged to explore how TES relates to student learning across extended periods. Finally, only one study distinguished subdimensions of TES (i.e., emotional climate, teacher sensitivity, and regard for student perspectives). Future empirical research could further investigate these subdimensions to clarify the specific mechanisms through which TES contributes to student learning.

## Figures and Tables

**Table 1:** Coding rules and sub-items of moderators.

Moderators	Items	Coding	Sub-Items
Participant educational level	Primary school	PS	
	Secondary school	SS	Junior high school, senior high school
	Higher education	HE	Undergraduate level, postgraduate level
Geographic region	East Asian context	EA	China, Vietnam
	Middle Eastern context	ME	Iran, Turkey, Israel
	Western context	W	USA, Norway, Australia, Germany, Belgium, Italy, Slovenia, Greece
Type of student learning outcome	Academic self-efficacy	ASE	
	Student engagement	SE	Academic engagement, emotional engagement, interaction engagement, behavioral engagement, agentic engagement, social engagement, cognitive engagement, learning engagement, academic adjustment, homework completion
	Academic achievement	AA	Test score, course grade, GPA

**Table 2:** Effect size and heterogeneity test result.

Number of Effect Size	Point Estimate	95% CI Lower Limit	95% CI Upper Limit	Q-Value	df (Q)	*p*-Value	I^2^	Tau^2^
83	0.406	0.368	0.443	2995.815	82	<0.001	97.263	0.042

**Table 3:** Results of three learning outcome dimensions.

Learning Outcomes	Effect Size and 95% Interval				Heterogeneity			
	k	r	Lower Limit	Upper Limit	Q	df (Q)	*p*-Value	I^2^
Academic self-efficacy (ASE)	18	0.463 ***	0.385	0.533	597.039	17	<0.001	97.153
Student engagement (SE)	42	0.456 ***	0.405	0.503	1320.651	41	<0.001	96.895
Academic achievement (AA)	23	0.259 ***	0.197	0.319	456.365	22	<0.001	95.179
Total between					29.253	2	<0.001	

Note: *** *p* < 0.001 (same below); k = number of effect sizes.

**Table 4:** Results of moderators.

Moderator	Effect Size and 95% Interval			Test of Null (2-Tail)		Heterogeneity		
	k (Effect Sizes)	Point Estimate	95%Cl	Z-Value	*p*-Value	Q-Value	df (Q)	*p*-Value
Mixed effect analysis								
Participant educational level								
PS	9	0.319	[0.228, 0.405]	6.565	<0.001	175.962	8	0.000
SS	39	0.346	[0.325, 0.417]	12.445	<0.001	1250.509	52	0.000
PS&SS	4	0.286	[0.239, 0.332]	11.367	<0.001	4.275	3	0.233
HE	29	0.516	[0.455, 0.572]	14.026	<0.001	770.449	28	0.000
SS&HE	2	0.476	[−0.138, 0.826]	1.546	0.122	66.780	1	0.000
Total between						33.814	4	0.000
Geographic region								
EA	50	0.429	[0.378, 0.478]	14.695	<0.001	2187.310	49	0.000
ME	6	0.334	[0.200, 0.455]	4.712	<0.001	97.432	5	0.000
W	26	0.376	[0.308, 0.440]	10.048	<0.001	682.349	25	0.000
W&EA	1	0.410	[0.320, 0.492]	8.254	<0.001	0.000	0	1.000
Total between						2.902	3	0.407
Discipline								
chemistry	1	0.207	[0.174, 0.239]	12.140	<0.001	0.000	0	1.000
education	2	0.474	[0.357, 0.577]	7.121	<0.001	5.621	1	0.018
language	23	0.480	[0.410, 0.545]	11.721	<0.001	682.283	22	0.000
literature	1	0.170	[0.118, 0.221]	6.335	<0.001	0.000	0	1.000
math	17	0.358	[0.288, 0.424]	9.392	<0.001	549.006	16	0.000
PE	3	0.394	[0.175, 0.577]	3.402	0.001	52.269	2	0.000
science	2	0.461	[0.379, 0.536]	9.812	<0.001	3.253	1	0.071
N/A	34	0.383	[0.318, 0.444]	10.698	<0.001	988.182	33	0.000
Total between						108.596	7	0.000
Learning mode								
Offline	77	0.400	[0.360, 0.437]	18.047	<0.001	2714.422	76	0.000
Online/blended	6	0.485	[0.290, 0.642]	4.490	<0.001	237.768	5	0.000
Total between						0.785	1	0.376
Student type								
General S	74	0.413	[0.372, 0.452]	17.877	<0.001	2834.296	73	0.000
Special S	9	0.342	[0.236, 0.441]	5.998	<0.001	114.241	8	0.000
Total between						1.661	1	0.198

## Data Availability

The raw data supporting the conclusions of this article are available at https://doi.org/10.7910/DVN/FLVATE. The archived dataset contains the original extracted effect sizes before within-study aggregation. The statistical analyses reported in this article were performed using aggregated effect sizes derived from these data following the procedures described in the Section 3.

## Abbreviations

The following abbreviations are used in this manuscript:

TES	Teacher Emotional Support
SDT	Self-Determination Theory
PS	Primary school
SS	Secondary school
HE	Higher education
EA	East Asian context
ME	Middle Eastern context
W	Western context
ASE	Academic self-efficacy
SE	Student engagement
AA	Academic achievement
N/A	Not applicable

## Appendix A. Coding Table

**Table:**

No	Study Name	Sample Size	Educational Level	Geographic Region	Discipline	Type of Learning Indicator	Student Type	Learning Mode	Correlation Coefficient
1	(Gu & Chen, 2018)	329	SS	EA	Math	AA	GP	offline	0.138
		329	SS	EA	Language	AA	GP	offline	0.111
2	(X. Chen et al., 2018)	824	SS	EA	N/A	AA	GP	offline	0.13
3	(Teng et al., 2017)	606	PS	EA	N/A	AA	SP	offline	0.25
4	(Yao & Yan, 2022)	445	HE	EA	N/A	AA	GP	online	0.82
5	(J. Zhang et al., 2019)	3012	PS	EA	Math	AA	GP	offline	0.17
6	(Zhu, 2021)	2613	SS	EA	Math	AA	GP	offline	0.28
7	(W. Li & Bai, 2018)	331	SS	EA	N/A	AA	GP	offline	0.167
8	(Alhassan et al., 2025)	305	SS&HE	W	N/A	SE	SP	offline	0.693
9	(Fraser et al., 2024)	379	PS&SS	W	N/A	AA	SP	offline	0.26
		368	PS&SS	W	N/A	AA	SP	offline	0.24
		635	PS&SS	W	N/A	AA	GP	offline	0.27
10	(Ginns et al., 2018)	2002	SS	W	N/A	ASE	GP	offline	0.52
		2002	SS	W	N/A	SE	GP	offline	0.42
11	(Mireles-Rios et al., 2020)	386	SS	W	N/A	AA	SP	offline	0.22
		397	SS	W	N/A	AA	SP	offline	0.33
12	(Peng et al., 2025)	1874	SS	EA	N/A	SE	SP	offline	0.22
13	(Zhu & Kaiser, 2022)	2613	SS	EA	Math	AA	GP	offline	0.28
		2613	SS	EA	Math	ASE	GP	offline	0.44
14	(H. Dong, 2025)	364	HE	EA	Language	SE	GP	offline	0.659
15	(Kashy-Rosenbaum et al., 2018)	1641	SS	W	N/A	AA	GP	offline	0.19
16	(X. Li et al., 2024)	415	HE	EA	Language	SE	GP	offline	0.482
17	(Y. Yang et al., 2024)	632	SS	EA	Math	SE	GP	offline	0.513
		632	SS	EA	Math	ASE	GP	offline	0.53
18	(Affuso et al., 2023)	201	SS	W	N/A	AA	GP	offline	0.17
		201	SS	W	N/A	ASE	GP	offline	0.31
		218	SS	W	N/A	ASE	GP	offline	0.36
19	(Böheim et al., 2020)	266	SS	W	N/A	SE	GP	offline	0.31
		266	SS	W	N/A	AA	GP	offline	0.17
20	(Cheng et al., 2023)	1365	PS	EA	Literature	AA	GP	offline	0.17
		1365	PS	EA	Math	AA	GP	offline	0.17
21	(Chiu, 2021)	426	SS	EA	Math	SE	GP	online	0.39
22	(Duan et al., 2024)	827	HE	EA	N/A	ASE	GP	offline	0.672
23	(M. Dong et al., 2025)	1371	SS	EA	Physical Education	SE	GP	offline	0.216
24	(Engels et al., 2020)	725	SS	W	N/A	SE	GP	offline	0.21
25	(Fan, 2024)	1429	HE	EA	Language	SE	GP	online	0.431
26	(Gao et al., 2024)	712	HE	EA	Language	SE	GP	offline	0.397
27	(Garcia-Reid et al., 2015)	141	SS	W	N/A	SE	SP	offline	0.44
28	(Garcia-Reid et al., 2015)	226	SS	W	N/A	SE	SP	offline	0.35
29	(Gong & Xu, 2024)	556	HE	EA	N/A	ASE	GP	offline	0.49
30	(Q. Guo et al., 2025)	406	HE	EA	Physical Education	ASE	GP	offline	0.544
		406	HE	EA	Physical Education	SE	GP	offline	0.405
31	(W. Guo et al., 2025)	414	HE	EA	N/A	ASE	GP	offline	0.359
		414	HE	EA	N/A	SE	GP	offline	0.424
32	(L. He et al., 2024)	450	HE	EA	Language	ASE	GP	offline	0.475
		450	HE	EA	Language	SE	GP	offline	0.722
33	(Jia & Cheng, 2024)	362	HE	W&EA	N/A	SE	GP	offline	0.41
34	(Kitsantas et al., 2021)	4978	SS	W	Math	ASE	GP	offline	0.2
35	(Kong et al., 2025)	614	SS	EA	N/A	SE	GP	online	0.42
		614	SS	EA	N/A	ASE	GP	online	0.24
36	(Kosir & Tement, 2014)	816	PS&SS	W	N/A	AA	GP	offline	0.34
37	(M. Li, 2024)	313	HE	EA	Language	SE	GP	offline	0.841
38	(T. Li, 2024)	567	HE	EA	Language	SE	GP	offline	0.57
39	(Liang et al., 2025)	759	HE	EA	N/A	SE	GP	offline	0.466
40	(Q. Liu et al., 2023)	466	HE	EA	Language	SE	GP	offline	0.564
		466	HE	EA	Language	ASE	GP	offline	0.423
41	(Ma, 2025)	2610	SS	EA	Language	SE	GP	offline	0.582
42	(Moustakas et al., 2025)	518	SS	W	Math	AA	GP	offline	0.18
43	(Patrick et al., 2007)	602	PS	W	Math	ASE	GP	offline	0.45
		602	PS	W	Math	AA	GP	offline	0.27
44	(Huangfu et al., 2024)	3344	SS	EA	Chemistry	SE	GP	offline	0.207
45	(Romano et al., 2021)	205	SS	W	N/A	SE	GP	offline	0.54
46	(Sadoughi & Hejazi, 2021)	435	HE	ME	Language	SE	GP	offline	0.47
47	(Sadoughi & Hejazi, 2023b)	384	HE	ME	Language	SE	GP	offline	0.19
48	(Sadoughi & Hejazi, 2023a)	295	SS&HE	ME	Language	SE	GP	offline	0.18
49	(Sakiz, 2017)	633	PS	ME	Science	ASE	GP	offline	0.42
		633	PS	ME	Science	SE	GP	offline	0.5
50	(Sakiz et al., 2012)	317	SS	W	Math	ASE	GP	offline	0.55
51	(Shen et al., 2024)	464	HE	EA	Education	SE	GP	offline	0.53
52	(Skaalvik et al., 2015)	823	SS	W	Math	ASE	GP	offline	0.635
53	(L. Sun et al., 2025)	213	HE	EA	N/A	SE	GP	online	0.425
54	(Tran et al., 2025)	642	HE	EA	Education	SE	GP	offline	0.418
55	(Tvedt et al., 2021)	1379	SS	W	N/A	SE	GP	offline	0.59
56	(Vo et al., 2024)	230	HE	EA	Language	SE	GP	offline	0.326
57	(Weyns et al., 2018)	586	PS	W	N/A	SE	GP	offline	0.44
58	(Wu et al., 2024)	1138	SS	EA	Math	SE	GP	offline	0.405
59	(Wu et al., 2025)	230	SS	EA	Math	AA	GP	offline	0.334
60	(Yin & Luo, 2024)	1361	HE	EA	Language	SE	GP	offline	0.222
61	(W. Zhang & Hu, 2025a)	2633	HE	EA	Language	SE	GP	offline	0.598
62	(W. Zhang & Hu, 2025b)	314	SS	EA	Language	SE	GP	offline	0.412
63	(M. Zhou et al., 2025)	335	HE	EA	Language	ASE	GP	offline	0.544
		335	HE	EA	Language	SE	GP	offline	0.544
64	(Y. Zhang et al., 2024)	1506	SS	EA	Language	SE	GP	offline	0.39

Note: Student engagement (SE) was coded as a single category. When multiple effect sizes were reported within the same study (e.g., different engagement dimensions or teacher emotional support dimensions), they were aggregated into a single study-level effect size prior to the matter-analysis to account for within-study dependence. GP = general population, SP = specific population (e.g., immigrant, ethnic minority, or left-behind students).
